# Prevalence of body-focused repetitive behaviors in three large medical colleges of karachi: a cross-sectional study

**DOI:** 10.1186/1756-0500-5-614

**Published:** 2012-11-01

**Authors:** Efaza Umar Siddiqui, Syed Saad Naeem, Haider Naqvi, Bilal Ahmed

**Affiliations:** 1 MSc Epidemiology and Biostatistics, Aga Khan University, Karachi, 74800, Pakistan

**Keywords:** BFRBs, Dermatillomania, Trichotillomania, Onychophagia

## Abstract

**Background:**

Body-focused repetitive behaviors (BFRBs) that include skin picking (dermatillomania), hair pulling (trichotillomania) and nail biting (onychophagia), lead to harmful physical and psychological sequelae.

The objective was to determine the prevalence of BFRBs among students attending three large medical colleges of Karachi. It is imperative to come up with frequency to design strategies to decrease the burden and adverse effects associated with BFRBs among medical students.

**Methods:**

A cross-sectional study was conducted among 210 students attending Aga Khan University, Dow Medical College and Sind Medical College, Karachi, in equal proportion. Data were collected using a pre tested tool, “Habit Questionnaire”. Diagnoses were made on the criteria that a student must be involved in an activity 5 times or more per day for 4 weeks or more. Convenience sampling was done to recruit the participants aged 18 years and above after getting written informed consent.

**Results:**

The overall prevalence of BFRBs was found to be 46 (22%). For those positive for BFRBs, gender distribution was as follows: females 29 (13.9%) and males 17 (8.1%). Among these students, 19 (9.0%) were engaged in dermatillomania, 28 (13.3%) in trichotillomania and 13 (6.2%) in onychophagia.

**Conclusions:**

High proportions of BFRBs are reported among medical students of Karachi. Key health messages and interventions to reduce stress and anxiety among students may help in curtailing the burden of this disease which has serious adverse consequences.

## Background

Body-focused repetitive behaviors (BFRBs) refer to a group of behaviors that include skin picking (dermatillomania), hair pulling (trichotillomania) and nail biting (onychophagia), which result in physical and psychological difficulties [1]. These behaviors for some individuals are simply referred to as nervous habits [2]. However, these nervous habits become problematic when they interfere with the person’s everyday functioning. When these BFRBs cross this line, then they are classified as Impulse Control Disorders.

BFRBs most often begin in late childhood or adolescence. They are among the most poorly understood, misdiagnosed, and under treated groups of disorders. The key factor underlying BFRBs is difficulty resisting the urge or impulse to perform a certain behavior that causes a degree of relief. The behavior continues because the BFRB results in a more pleasant state therefore, it is negatively reinforced.

Prevalence estimates indicates that such behaviors are quite common among students. Nail biting of 2 times or more per week was reported among 63.6% students in United States of America [2]. Another study from USA, using the stringent criteria of 5 times or more per week stated 21.8% students engaged in mouth, lips or cheeks chewing and 10.1% engaged in habitual nail biting [3]. A recent survey of college students found that 13.7% of the sample endorsed in at least one repetitive behavior that occurred more than five times per day for at least 4 weeks and produced some type of psychological or physical disruption of functioning [4]. Based on questionnaire screenings, a lifetime ICD rate of 3.5% was found in college students of Germany [5]. Very limited literature and no prevalence estimates regarding BFRBs could be found from Asian countries including Pakistan.

The treatment for BFRBs may include a combination of psychotropic medications and cognitive behavioral therapy (CBT). CBT often involves Habit Reversal Training and Exposure and Response Prevention (aka Exposure and Ritual Prevention).

Since limited literature from our part of the world is available on body-focused repetitive behaviors and its impact on medical students and their lifestyles, it is imperative to spread awareness regarding them and find out ways and means to prevent them. It is therefore essential to come up with frequency to design strategies to decrease the prevalence of BFRBs and thus prevent their adverse effects among medical students.

We thus aimed to determine the prevalence of BFRBs among students attending three large medical colleges of Karachi.

## Methods

The study was conducted in three large medical colleges of Karachi: The Aga Khan University, Dow Medical College and Sindh Medical College. Students from Karachi as well as interior and foreign attend these institutes; hence provide better representation from different socio economic classes and cultures. The combined strength of student of the three colleges was approximately 3000. The study was conducted in July 2010.

The investigation was a cross-sectional study. Students were recruited in equal proportion from each college. A self-administered pre tested questionnaire was used for data collection. Data was collected by trained medical students. The study was approved by the Ethical Review Committee of Aga Khan University and Dow Medical College. Written informed consent was obtained from each student and an explanation of the purposes of the research was provided to them.

Eligibility criteria to participate in the study comprised of registered medical students aged 18 and above. However students less than 18 years and not willing to consent to participate in this study were excluded.

A d “Habit Questionnaire” was used for gathering information on repetitive behaviors. Habit is a brief five items, self-report questionnaire that provides a standardized assessment of the frequency and duration of BFRBs.

The Habit Questionnaire has been found to possess moderate test–retest reliability of 0.69 of diagnosis of BFRB, p<0.001 [4].

Participants were asked to indicate if they engage in the following repetitive behaviors: hair pulling, hair manipulation, nail biting, skin biting and mouth chewing. For each repetitive behavior, participants who acknowledged engaging in the specified behavior were asked about the frequency of the behavior (i.e., fewer or greater than five times per day) and the duration of the behavior (i.e., less than 4 weeks or longer than 4 weeks). Participants were also asked to specify if the behavior caused impairment, which was defined as (1) interference with daily functioning, (2) resultant injuries with possible permanent scarring or damage, (3) medical attention as a result of the behavior or (4) recommendation to cease the behavior by a medical professional. To rule out conditions that commonly co-occur with BFRBs, the final section of the Habit Questionnaire asked participants to indicate if they had ever been diagnosed with obsessive–compulsive disorder, Tourette’s syndrome, autism, Asperger’s syndrome or a developmental disability.

To meet the criteria for a BFRB, the participants needed to answer “yes” to engaging in at least one repetitive behavior five or more times per day, and the behavior had to be present for at least 4 weeks. Participants were also needed to report that the particular behavior interferes with functioning, caused an injury, caused him or her to seek medical attention or elicited a recommendation to stop the behavior by a medical professional.

Our focus was on three behaviors: Trichotillomania (including ‘hair pulling’ and ‘hair manipulation’), Onychophagia (including ‘nail biting’) and Dermatillomania (including ‘chew mouth lips or cheek’ and ‘bite on any other areas’).

### Statistical analysis

A sample size of at least 210 was required to estimate the prevalence of BFRBs among medical students assuming 14% prevalence figure of the Pakistani population, along with 80 percent power, 0.05 significance level, 5 percent bond on error and 10% adjustment for non-response rate. Convenience sampling was used by getting the questionnaires filled during regular college hours from students at four locations in the medical college: the lecture halls, laboratories, library and canteen. Questionnaires were filled on consecutive days until the required sample size was achieved.

Data was entered and analyzed using Statistical Package for the Social Sciences (SPSS) version 13.0. Initially descriptive statistics, frequencies and proportions were generated. Continuous variables were analyzed by t – test and categorical by chi-square or Fisher exact, where appropriate.

## Results

The prevalence of BFRBs among medical students of Karachi was found to be 46 (22%). For those positive for BFRBs, gender distribution was as follows: females 29 (13.9%) and males 17 (8.1%). The average age of participants was 21.5 ranging from 18–27 (refer Table 1). Among these students, 19 (9.0%) were engaged in dermatillomania, 28 (13.3%) in trichotillomania and 13 (6.2%) in onychophagia (refer Table 2).

**Table 1:** Baseline characteristics of participants

Age	Mean=21.5	Range=18-27
gender	males (55)	females (155)

**Table 2:** BFRBs characteristics among medical students in Karachi

**Variable**	**males**	**females**
**Hair Pulling**		
Yes	9	19
No	46	136
< 5 times a day	7	12
≥ 5 times a day	2	7
< 4 weeks	1	1
4 weeks to 12 months	1	0
≥ 12 months	7	18
noticeable hair loss	3	5
interfere day to day activity	2	3
behavior cause injury	1	2
cause permanent scar or damage	3	1
seek medical attention	1	2
medical professional suggested you to stop it	1	2
behavior under the influence of alcohol or drugs	0	2
**Hair Manipulation**		
Yes	31	34
No	24	121
< 5 times a day	16	23
≥ 5 times a day	15	11
< 4 weeks	4	5
4 weeks to 12 months	7	6
≥ 12 months	20	23
noticeable hair loss	5	8
interfere day to day activity	4	3
behavior cause injury	1	2
cause permanent scar or damage	1	1
seek medical attention	2	3
medical professional suggested you to stop it	1	5
behavior under the influence of alcohol or drugs	1	0
**Nail Biting**		
Yes	11	36
No	44	119
< 5 times a day	7	27
≥ 5 times a day	4	9
< 4 weeks	0	3
4 weeks to 12 months	0	8
≥ 12 months	11	25
interfere day to day activity	4	5
behavior cause injury	2	6
cause permanent scar or damage	2	3
seek medical or dental attention	2	3
medical professional suggested you to stop it	5	6
behavior under the influence of alcohol or drugs	0	1
**Chew Mouth, Lips or Cheeks**		
Yes	23	51
No	32	104
< 5 times a day	17	36
≥ 5 times a day	6	15
< 4 weeks	3	8
4 weeks to 12 months	3	5
≥ 12 months	17	38
interfere day to day activity	1	1
behavior cause injury	5	10
cause permanent scar or damage	2	1
seek medical attention	1	0
medical professional suggested you to stop it	3	5
behavior under the influence of alcohol or drugs	0	0
**Bite on Other Areas**		
Yes	5	9
No	50	146
< 5 times a day	3	8
≥ 5 times a day	2	1
< 4 weeks	0	0
4 weeks to 12 months	1	3
≥ 12 months	4	6
interfere day to day activity	0	2
behavior cause injury	3	3
cause permanent scar or damage	0	1
seek medical attention	1	0
medical professional suggested you to stop it	2	1
behavior under the influence of alcohol or drugs	1	1

As discussed earlier, to meet criteria for BFRBs, student must report engaging in an activity more than five times a day for at least 4 weeks or more. Table 3 clearly shows that majority of the students that were involved in an activity repeated it less than five times per day, rendering them ineligible to be labeled as being involved in BFRBs. Also, far less number of students engaging in a BFRB said to have been involved in it for 4 weeks to 12 months while majority fell into 12 months or more category: 7 out of 9 males and 18 out of 19 females for hair pulling; 20 out of 31 males and 23 out of 34 females for hair manipulation; 11 out of 11 males 25 out of 36 females for nail biting; 17 out of 23 males and 38 out of 51 females for chewing mouth, lips or cheeks and 4 out 5 males and 6 out of 9 females for biting other areas.

**Table 3:** Prevalence and gender wise distributions of BFRBs

	**Males**	**Females**	**Total**	**Total %**	**p-values**
BFRBs +ve	17	29	46	22%	0.05
Dermatillomania	6	13	19	9.0%	0.57
Trichotillomania	14	14	28	13.3%	< 0.00
Onychophagia	4	9	13	6.2%	0.69

Many of the students being engaged in an activity also reported that that activity resulted in noticeable hair loss, interfered with day to day activity, caused injury, permanent scar or damage or made them seek medical attention. Few of them reported that a medical professional suggested them to stop the behavior while very few said that the behavior occurred under the influence of alcohol or other drugs (for details refer Table 3). Besides this, as shown in the previous studies, we did obtain the same test retest reliability.

## Discussion

BFRBs are not uncommon among medical students of Karachi. It is therefore imperative to identify the prevalence associated with BFRBs to design interventions to curtail the burden.

Disorder-specific rates in our study ranged from 6.2%-13.3%, which is in agreement with the rates of 12.8% and 10.0% of skin picking and nail biting respectively according to a study conducted in the U.S. [6]. Our study also found greater occurrence among females than males, a finding consistent with previous studies [1,7,8]. Literature shows that stressful conditions tend to trigger BFRBs but may not be necessarily associated. Thus this higher prevalence may indicate that females are more prone to stressful conditions and thus tend to get engaged more frequently in BFRBs than males.

As mentioned above, stress may trigger BFRBs. Besides that, other reasons such as socioeconomic factors may also be the cause of stress and anxiety among different sets of students. But since 1) no literature is available proving any such facts, 2) our sample is limited and has no control population and 3) ours is a cross-sectional study which cannot establish cause and effect relationship, therefore we can’t come up with a valid conclusion in this regard.

Overall prevalence of BFRBs in our study was found to be much higher than previous surveys for e.g., according to a study, 13.7% of college students endorsed at least one repetitive behavior that occurred more than five times per day for at least 4 weeks and produced some type of psychological or physical disruption of functioning [4]. Another source gives a lifetime ICD rate of 3.5% in college students [5] while according to a study conducted in United States, 17.1% subjects met criteria for a current ICD [6]. Disorder-specific rates are far greater than other studies, such as 2.7-6.4% of skin picking, mouth chewing and nail biting [4] and disorder-specific rates of 0.4-1.2%, except for trichotillomania, which was 0% [5].

Research suggests that skin picking occurs in people with a mean age of onset of around 15 years [9] but our study comprised of medical students aged 18 and above, with those indulging in skin picking ranging from 19–26 years with a mean of 22.5 years. Previous studies also identified prevalence of dermatillomania to be approximately 3.8-4.6% of college students [8,10] 5.4% of a community sample, 4% of college students and 2% of patients seen in a dermatology clinic [8,11-14], and 1.4%, 4.6% and 3.8% by Nancy J. Keuthen on various occasions [8,9]. It should be noted here that all these figures are far lower than the prevalence of dermatillomania in our study population that is 9.0%, possibly depicting a greater influence of stress and anxiety on medical students [15].

Expression of repetitive behaviors has been well documented in the trichotillomania literature. Prevalence estimates indicate a frequency of 2.5% of young adults [16], being quite lower than the prevalence of trichotillomania found in our study, that is 13.3%. This might be due to inclusion of ‘hair manipulation’ in addition to ‘hair pulling’ in our study.

It is interesting to note that medical students of Karachi were found to indulge the most into trichotillomania, followed by dermatillomania and the least prevalent behavior was onychophagia which is contrary to literature present. This might be due to the fact that in our study, Trichotillomania and Dermatillomania both comprised of two behaviors whereas Onychophagia comprised of nail biting alone, as has been mentioned above.

In one of the few studies to address the issue of BFRBs, college students were categorized as having a repetitive behavior (habit) if the student reported engaging in a behavior two or more times per week [2]. Using this relatively lenient criterion, Hansen et al. found that nail biting occurred in 63.6% of the sample. A subsequent study used more stringent criteria for identifying repetitive behaviors in college students. Stating that the repetitive behavior had to occur at least five times per day to be classified as a habit, it was found that 21.8% of the sample engaged in habitual chewing on mouth, lips, or cheeks and 10.1% engaged in habitual nail biting [3].

As discussed earlier, BFRBs may produce a variety of physical sequelae. Thus, to accurately ascertain the extent to which BFRBs are an actual diagnosable problem, not only must data be collected on how frequently these behaviors occur in an individual but also and more importantly the extent to which these behaviors produce some type of impairment be considered. Unfortunately, the Hansen et al. (1990) and Woods et al. (1996) did not incorporate this variable into their operational definitions when determining the prevalence of BFRBs. The tool that we have used for our study, the ‘Habit Questionnaire’ follows criteria that cover all factors and variables associated with BFRBs and thus give a better estimate of its prevalence with greater accuracy. Even after following such strict criteria, prevalence in our study was quite high, pointing towards the fact that medical students of Karachi are greatly prone to such negative behaviors.

There are a number of aspects of our study which limit conclusions. Due to excessive workload on medical students, the specifications of questionnaires might not have been accurately filled or due to extra stress on students having their exam season and vice versa, their BFRBs might have been affected which might have affected the overall results. Also, since ours is a cross-sectional study, cause and effect relationship for the identified associated factors could not be established. Because we have carried out this study among medical students only, we cannot give any absolute predictions of prevalence among the general population. Besides that, we cannot comment on the age and gender distribution of BFRBs since medical students fall into a particular age group only and also because the majority of students at the above mentioned study setting were females, it may have affected gender distribution of such behaviors. Also, we have not considered co-morbidities of different behaviors which limit conclusions.

This study offers a number of avenues for future research. Additional research should be conducted to establish prevalence rates among different populations including children, adolescents and elderly, and among populations with cultural differences. It may also be useful to examine the general phenomenology of BFRBs among typically developing persons, including possible co-occurring psychological symptoms such as anxiety or negative affective states. Studies should also be conducted to find out the factors associated with BFRBs and its various possible consequences. Doing so may elucidate important components of treatment and methods to avoid engaging into such behaviors that are so physically and socially disadvantageous.

## Conclusions

The above facts clearly indicate a comparatively higher prevalence of BFRBs among medical students of Karachi than other populations, supporting the fact that these students are a victim of great stress and anxiety in their daily lives. High occurrence of BFRBs among them can be greatly detrimental and thus there is an utter need to spread awareness regarding these behaviors to save the students from its negative impacts. Key health messages and interventions to reduce stress and anxiety among students may help in curtailing the burden of this disease which has serious adverse consequences.

## Abbreviations

BFRBs: Body Focused Repetitive Behaviors; CBT: Cognitive Behavioral Therapy; ICD: Impulse Control Disorder; SPSS: Statistical Package for the Social Sciences.

## Competing interests

We do not have any competing interests to declare.

## Authors’ contributions

EUS came up with the idea, did literature review, made the proposal, did data collection, entered data into Statistical Package for the Social Sciences, analyzed it and wrote the paper. SSN helped with literature review, did data collection, entered data into Statistical Package for the Social Sciences and analyzed it. HN’s role was as a consultant psychiatrist, BA supervised the overall conduction of study. All authors read and approved the final manuscript.

## Authors’ information

EUS and SSN are final year medical students at Dow Medical College, Dow University of Health Sciences, Karachi, Pakistan. HN = MBBS, FCPS, BA MSc.
